# Antioxidant effects of phenolic compounds in through the distillation of *Lonicera japonica & Chenpi* extract and anti-inflammation on skin keratinocyte

**DOI:** 10.1038/s41598-023-48170-w

**Published:** 2023-11-28

**Authors:** Hun Hwan Kim, Se Hyo Jeong, Min Yeong Park, Pritam Bhangwan Bhosale, Abuyaseer Abusaliya, Hyun Wook Kim, Je Kyung Seong, Meejung Ahn, Kwang Il Park, Gon Sup Kim

**Affiliations:** 1 Research Institute of Life Science and College of Veterinary Medicine, Gyeongsang National University, Jinju, 52828 Republic of Korea; 2 Division of Animal Bioscience & Intergrated Biotechnology, Jinju, 52725 Republic of Korea; 3 Laboratory of Developmental Biology and Genomics, BK21 PLUS Program for Creative Veterinary Science Research, Research Institute for Veterinary Science, College of Veterinary Medicine, Seoul National University, Seoul, 08826 Republic of Korea; 4 Department of Animal Science, College of Life Science, Sangji University, Wonju, 26339 Republic of Korea

**Keywords:** Biochemistry, Molecular biology

## Abstract

The phenolic compounds in *Lonicera japonica & Chenpi* distillation extract (LCDE) were thoroughly examined for their antioxidant and anti-inflammatory properties. Phenolic compounds in LCDE were analyzed for five peaks using high-performance liquid chromatography (HPLC) combined with mass spectrometry (MS) and determined. Five phenolic compounds were identified from the samples and MS data. Ultrafiltration with LC analysis was used to investigate the ability of bioactive compounds to target DPPH. As a result, it was confirmed that the major compounds exhibited a high binding affinity to DPPH and could be regarded as antioxidant-active compounds. In addition, the anti-inflammatory effect of LCDE was confirmed in vitro, and signal inhibition of anti-inflammation cytokines, MAPK and NF-kB pathways was confirmed. Finally, Molecular docking analysis supplements the anti-inflammatory effect through the binding affinity of selected compounds and inflammatory factors. In conclusion, the phenolic compounds of the LCDE were identified and potential active compounds for antioxidant and anti-inflammatory activities were identified. Additionally, this study will be utilized to provide basic information for the application of LCDE in the pharmaceutical and pharmaceutical cosmetics industries along with information on efficient screening techniques for other medicinal plants.

## Introduction

LCDE is a mixture of *Lonicera japonica*, *Taraxacum*, *Chenpi*, *Forsythia* and *Licorice*. It is used for acute mastitis and various purulent infections. *Lonicera japonica* contains various active substances such as saponin, flavonoids, tannins, alkaloids, and fatty acids in the outpost, and has excellent active oxygen removal, anti-inflammatory, antibacterial, antipyretic, soothing, and diuretic effects, so it can also be used to treat colds, body aches, food poisoning boils, immobility, and hyperlipidemia^1^. It is effective and is known to help prevent and treat various infections^1^. *Taraxacum* is rich in vitamins and minerals and is known to have anti-inflammatory properties^2^. The citrus peels, *Chenpi* stimulate blood circulation in capillaries to relieve stress on the skin surface, and are effective for skin diseases such as acne, suppuration, eczema, and itching^3^. *Forsythia* is known to have the effect of lowering fever and detoxifying^4^. *Licorice* harmonizes all medicinal materials to make the medicinal effect appear well and is known to have detoxification, hepatitis, hives, dermatitis, eczema, diuretic and anti-inflammatory effects^5^.

Inflammation is a defense process that causes fever, swelling, discomfort, and a variety of functional impairments in response to various diseases and injuries^6^. In severe circumstances, acute inflammation can cause severe harm and even death^7^. When acute inflammation progresses to chronic inflammation, various diseases such as cancer, atherosclerosis, osteoarthritis, and Alzheimer's disease might develop^8^. Controlling inflammation and inflammation-related diseases remains a significant challenge^9^. Additionally, anti-inflammatory medication research and development are key challenges in modern medicine^10^. Antioxidant activity protects cell membranes by removing free radicals and is closely related to anti-inflammatory mechanisms by inhibiting prostaglandins, a major inflammatory factor produced during metabolism^11,12^.

The skin is the primary interface between the body and the environment and provides the first line of defense against microbial and chemical agents^13^. Especially in skin inflammation, dermatitis (atopic, contact, and seborrheic) are the most common types. While contact dermatitis is defined by itching and inflammation of the skin as a result of contact with external substances, atopic dermatitis is an inflammatory condition brought on by hereditary influences on immune cells and proteins that make up the skin. An inflammatory skin condition known as seborrheic dermatitis affects regions with a lot of sebaceous gland^14,15^.

Oxidative stress and skin inflammation are one of the damages that the skin disease^16^. Mechanisms of inflammation and oxidative stress in skin diseases have already been identified^17,18^. However, it was unknown whether *Lonicera japonica & Chenpi* distillation extract (LCDE) treatment affects pro-inflammatory cytokines associated with the progression of skin damage in HaCaT cells^11^ and which selected compounds have the extent of antioxidant effect.

Five phenolic compounds were selected with LC–MS/MS, and the antioxidant effect due to the difference in HPLC-DPPH binding area value was confirmed. Additionally, investigated whether inflammation was suppressed by LCDE inducing inflammation in HaCaT cells with lipopolysaccharide (LPS) treated. Through these results, the phenolic compound having a significant effect on anti-inflammation in LCDE was bound to the related inflammatory protein using molecular docking, and the docking score was analyzed. These docking data and in vitro results suggest that phenolic compounds present and retained through the distillation extraction method of LCDE, a plant extract, have potent antioxidant and anti-inflammatory activities and can be useful cosmeceuticals for dermatitis. Our research method is differentiated from existing research methods that use literature searches and individual standards to confirm physiological activity. Existing methods not only cost a lot of charge and time, but also use standard substances, making it difficult to understand the competitive reactions of compounds contained in the extract. However, because our research method induces a competitive reaction for the compounds contained in the extract, we can quickly and accurately identify the most efficient compounds. This research method not only saves researchers time and charge, but also helps in selecting effective compounds among various compounds. Therefore, this study can be efficiently applied to the early screening stage of new drug development as well as information on effective ingredients among the ingredients contained in LCDE.

## Results

### Phenolic compounds separation and characterization in LCDE

**Figure 1:**
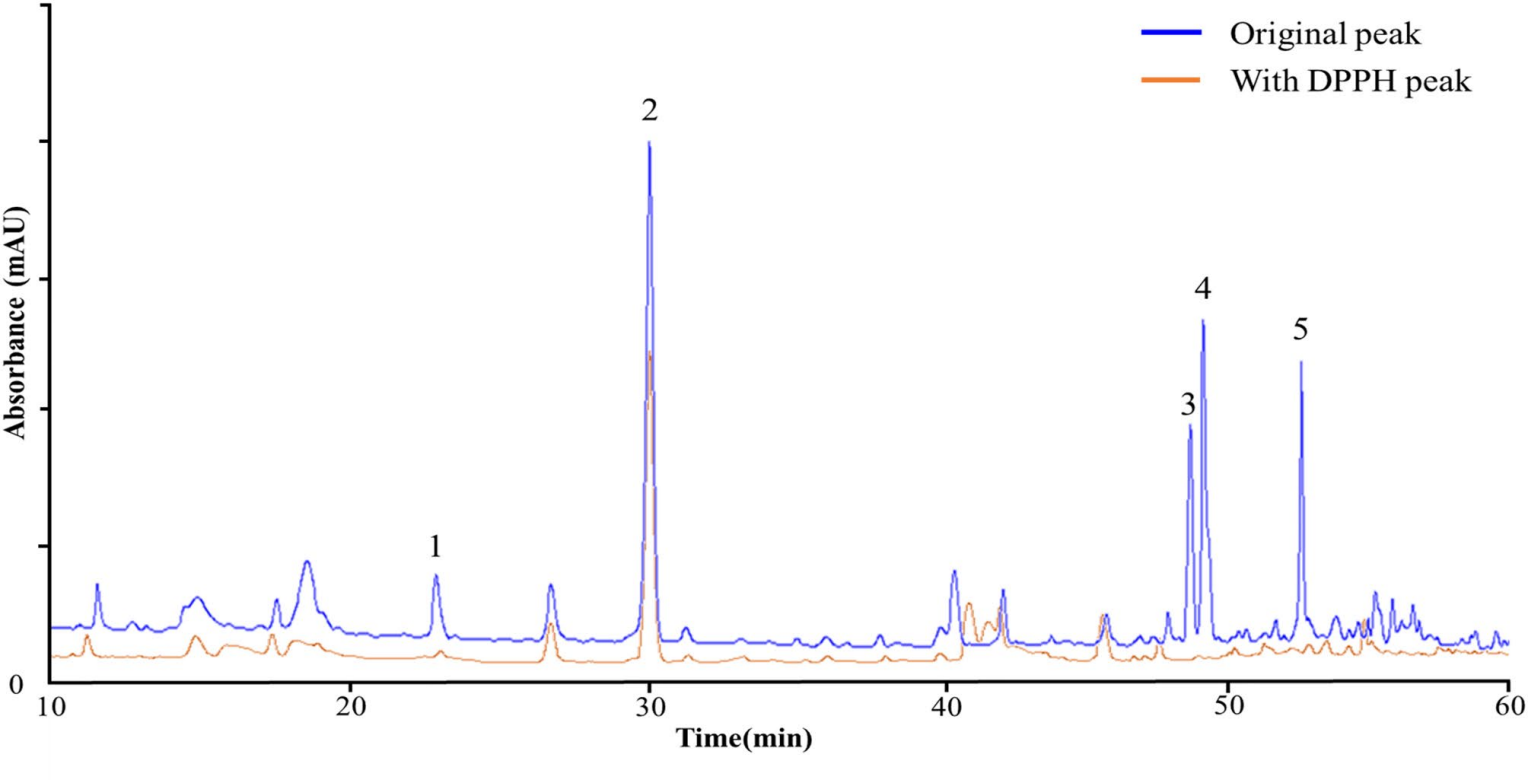
The HPLC chromatograms of the phenolic compounds in LCDE. The blue line is the original chromatogram at the beginning of the LCDE, while the orange line is the chromatogram after reaction with DPPH solution. The detected compounds at the 284 nm wavelength are sweroside (1), isoliquiritin (2), cardamonin (3), riboflavin (4) and arctigenin (5).

**Figure 2:**
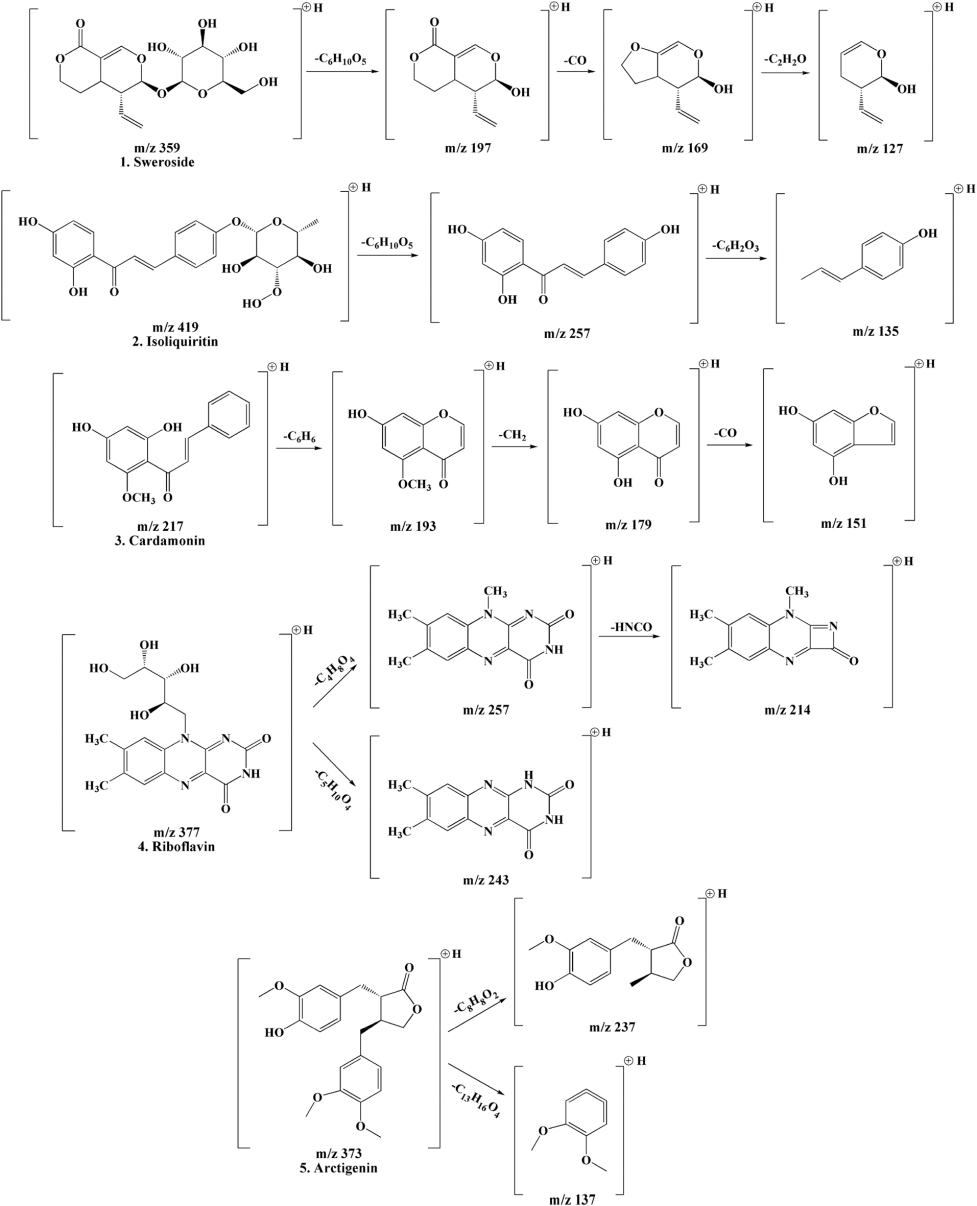
Fragmentation scheme of the phenolic compounds contained in LCDE.

HPLC–MS/MS was used to analyze the compounds found in LCDE. A total of 5 peaks were identified based on HPLC retention time and UV–vis spectrum. 5 compounds were identified according to the peaks obtained by HPLC chromatograph at a wavelength of 284 nm (Fig. 1). The 5 phenolic compounds were identified as sweroside^19^, isoliquiritin^20^, cardamonin^21^, riboflavin^22^ and arctigenin^23^ based on fragmentation patterns. Table 1 provides a description of the mass spectrometry identification data based on reference compounds from published sources. The following physiologically active compounds are the outcomes that, depending on the environment or growing conditions of plants, can be different in various ways. The characterization of the identified phenolic compounds is the main focus of this study. Phenolic compounds were identified based on chemical ion peaks and mass patterns acquired by LC–MS/MS and contrasted with previously discovered literature data^19–23^. Based on LC–MS/MS data, the cleavage process of the compounds and shown in Fig. 2.

**Table 1:** The HPLC–MS/MS data of phenolic compounds from LCDE.

Peak no.	Rt (min)	Formula	Compound	UV max	[M + H]^+^	MS/MS
1	23.04	C_16_H_22_O_9_	Sweroside	245	359	197 (C_10_H_12_O_4_) [M + H-C_6_H_10_O_5_]^+^ 169 (C_9_H_12_O_3_) [M + H-C_6_H_10_O_5_-CO]^+^ 127 (C_7_H_10_O_2_) [M + H-C_7_H_10_O_7_-C_2_H_2_]^+^
2	30.24	C_21_H_22_O_9_	Isoliquiritin	370, 235	419	257 (C_15_H_12_O_4_) [M + H-C_6_H_10_O_5_]^+^
3	48.48	C_16_H_14_O_4_	Cardamonin	340	271	193 (C_10_H_8_O_4_) [M + H-C_6_H_6_]^+^ 179 (C_9_H_6_O_4_) [M + H-C_6_H_6_-CH_2_]^+^ 151 (C_8_H_6_O_3_) [M + H-C_7_H_8_-CO]^+^
4	48.92	C_17_H_20_N_4_O_6_	Riboflavin	440, 365	377	257 (C_13_H_12_N_4_O_2_) [M + H-C_4_H_8_O_4_]^+^ 243 (C_12_H_10_N_4_O_2_) [M + H-C_5_H_10_O_4_]^+^ 214 (C_12_H_11_N_3_O) [M + H-C_4_H_8_O_4_-HNCO]^+^
5	52.23	C_21_H_24_O_6_	Arctigenin	280, 230	373	237 (C_14_H_16_O_4_) [M + H-C_8_H_8_O_2_]^+^ 137 (C_8_H_8_O_2_) [M + H-C_13_H_16_O_4_]^+^

*Rt* retention time.

### Screening of Antioxidant phenolic compounds in LCDE

The antioxidant effect is typically confirmed using the DPPH radical scavenging activity assay, which is helpful for validating the antioxidant activity of complex chemicals found in natural products. In this study, DPPH-HPLC analysis was conducted to select potential antioxidant candidates present in LCDE. As shown in Fig. 1, various active compounds contained in LCDE react with DPPH and the peak decreases. The reaction of the compounds with DPPH is shown by the change in the peak area value in Table 2. Additionally, the difference between the peak area values before and after DPPH binding in the reaction with DPPH shows stronger radical scavenging activity.

**Table 2:** Screening of antioxidant potential of LCDE compounds.

Peak no.	Compound	Initial area	Area after DPPH reaction	Reactive area/(%)
1	Sweroside	118.37 ± 2.44^A^	11.63 ± 0.70^A^	106.73 ± 3.02^A^ (90.16 ± 0.75^B^)
2	Isoliquiritin	989.47 ± 9.45^E^	719.87 ± 7.85^B^	269.60 ± 6.78^B^ (27.25 ± 0.57^A^)
3	Cardamonin	252.57 ± 2.81^B^	12.53 ± 1.80^A^	240.03 ± 3.50^B^ (95.04 ± 0.72^C^)
4	Riboflavin	435.70 ± 5.35^D^	30.27 ± 1.91^A^	405.43 ± 6.16^C^ (93.05 ± 0.47^BC^)
5	Arctigenin	284.50 ± 4.79^C^	15.67 ± 1.31^A^	268.83 ± 3.64^B^ (94.50 ± 0.38^C^)

All values are mean ± SD (n = 3). ^A–D^Means with different superscripts in the same column are significantly different at *p* < 0.05 by Duncan’s multiple range tests.

In Table 2, the difference between the initial peak area value of each phenolic compound in LCDE and the area value following the DPPH reaction serves as evidence of the antioxidant impact. Riboflavin showed the highest change in area value at 405.43mAU, and the rate of change was also 93.05%, showing high DPPH binding capacity. Sweroside, cardamonin, and arctigenin showed high area change ratios of 90.16%, 95.04%, and 94.50%, respectively, but the area changes were 106.73mAU, 240.03mAU, and 268.83mAU, which were lower than those of riboflavin. Isoliquiritin had an area value change of 269.60, similar to cardamonin and arctigenin, but the area value change rate was low at 27.25%.

Due to the difference in DPPH binding ability of each phenolic compound, riboflavin, cardamonin, and arctigenin have high DPPH activity and high antioxidant activity. In addition, these results showed that all five compounds (sweroside, isoliquiritin, cardamonin, riboflavin, and arctigenin) had antioxidant effects and were selected as major compounds for LCDE.

### The effect of LCDE on the cell viability of HaCaT Cells

**Figure 3:**
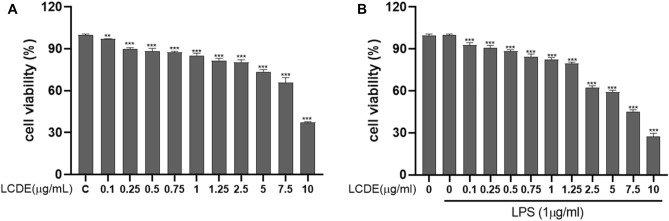
The effects of LCDE on HaCaT keratinocytes cell viability. Data were from three independent experiments. LCDE (0, 0.1, 0.25, 0.5, 0.75, 1, 1.25, 2.5, 5, 7.5 and 10 μg/mL) was treated by concentration, and then (**A**) the toxicity of LCDE to cells for 24 h were measured. (**B**) Cell viability when LPS and LCDE were treated with or without for 24 h. Comparison with LCDE and LPS treated group **p < 0.01; ***p < 0.001.

To determine the cytotoxicity of the LCDE, the 3-(3,4-dimethyl-thiazolyl-2)-2,5-diphenyl tetrazolium bromide (MTT) assay was carried out in HaCaT cells (Fig. 3). With or without 1 µg/mL of LPS, the LCDE treated HaCaT cells at concentration of 0, 0.1, 0.25, 0.5, 0.75, 1, 1.25, 2.5, 5, 7.5 and 10 μg/mL for 24 h. As a results, We found that the LCDE was non-toxic at 0.1 to 0.75 μg/mL (Fig. 3A). In Fig. 3B, even when inflammation was induced through LPS, there was no toxicity of the extract at the same concentration. As a result, the concentration judged to be the least cytotoxic was selected and used for follow-up research.

### Effects of LCDE on COX-2 and iNOS expression of LPS induced HaCaT cells

**Figure 4:**
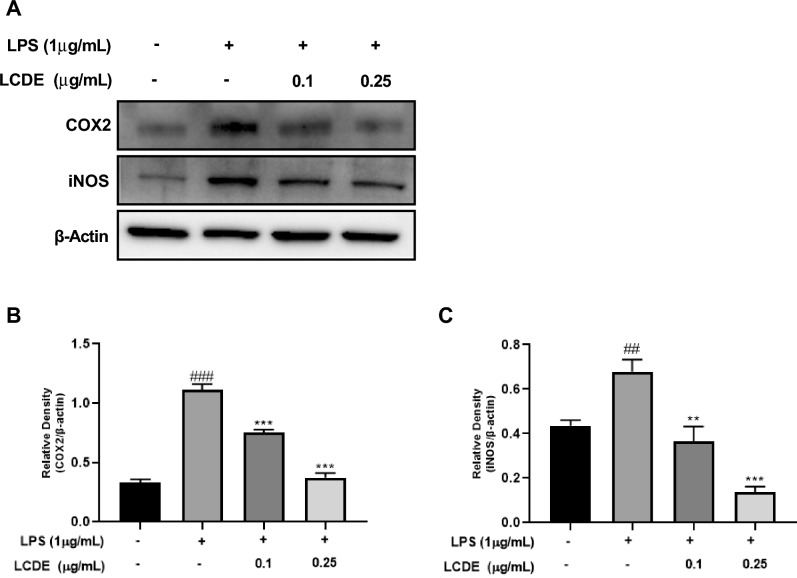
Effect of LCDE on COX-2 and iNOS expression in with or without LPS-stimulated HaCaT cells. HaCaT cells were treated with LCDE (0, 0.1 and 0.25 μg/mL) with or without LPS (1 μg/mL) for at 37 °C 24 h. (**A**) The expression of COX2 and iNOS was quantified by western blot analysis. (**B**) Relative density of COX-2 expression. (**C**) Relative density of iNOS expression. Comparison with LCDE and LPS treated group **p < 0.01; ***p < 0.001. Comparison with LPS treated group ^##^*p* < 0.01; ^###^*p* < 0.001.

NO (Nitric Oxide) is an inflammatory mediator that produced by iNOS and COX-2. Therefore, the downregulation of the inflammatory factors COX-2 and iNOS are important in regulating inflammation^24^. The anti-inflammatory effect was explored using western blot to evaluate the expression of iNOS and COX-2 proteins, and we discovered that LCDE decreased COX-2 and iNOS expression in a dose-dependent manner in HaCaT cells (Fig. 4).

### Inhibition of LPS-induced MAPKs pathways activation by LCDE

**Figure 5:**
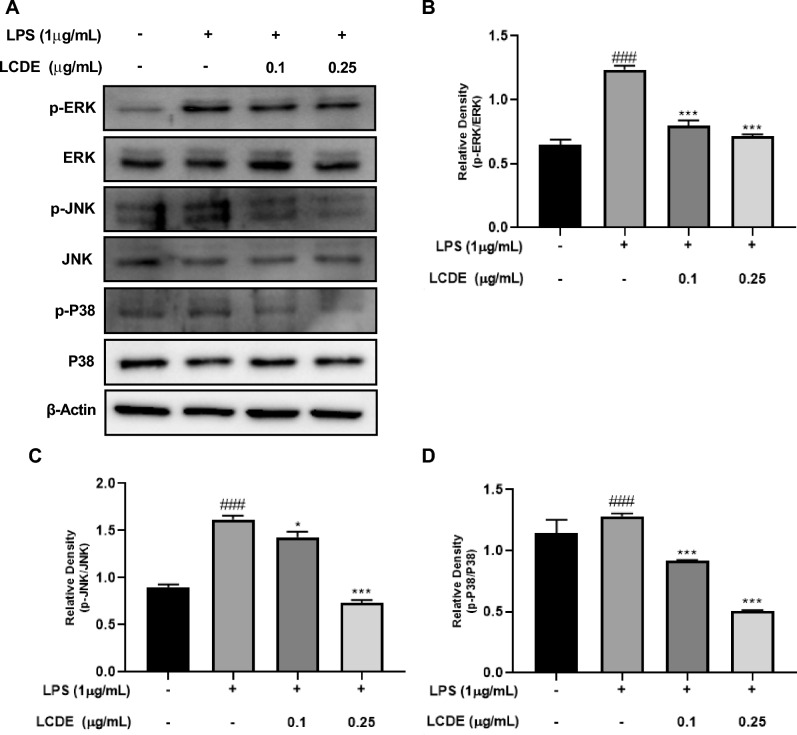
Effect of LCDE on with or without LPS induced MAPKs protein expression in HaCaT cells. HaCaT cells were treated with LCDE (0, 0.1 and 0.25 μg/mL) with or without LPS (1 μg/mL) for at 37 °C 24 h. (**A**) The expression of MAPKs was quantified by western blot analysis. (**B**) Expression of p-ERK affected by LCDE. (**C**) Expression of p-JNK affected by LCDE. (**D**) Expression of p-P38 affected by LCDE. Comparison with only LPS *p < 0.05; ***p < 0.001. Comparison with LCDE and LPS treated group ^##^*p* < 0.01; ^###^ *p* < 0.001.

MAPKs (JNK, ERK, and P38) are present in the cytoplasm, but when activated by LPS, they are phosphorylated and translocate to the nucleus. LPS-treated cells expressed more JNK, p38, and ERK, as shown in Fig. 5. Co-treatment with LPS and LCDE, on the other hand, phosphorylation of MAPKs (JNK, ERK, and P38) are suppressed by the expression of these markers in a dose-dependent manner. These data suggest that LCDE has anti-inflammatory effects on LPS-stimulated HaCaT cells by modulating MAPK pathways.

### Inhibition of LPS induced NF-κB pathways activation by LCDE

**Figure 6:**
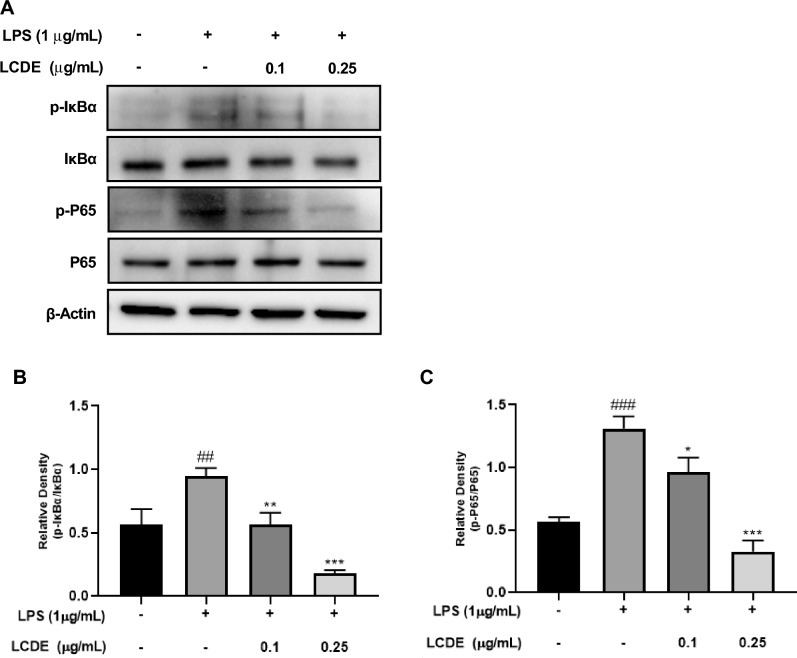
Effect of LCDE on with or without LPS induced protein expression of NF-κB in HaCaT cells. HaCaT cells were treated with LCDE (0, 0.1 and 0.25 μg/mL) with or without LPS (1 μg/mL) for at 37 °C 24 h. (**A**) The expression of NF-κB were quantified by western blot analysis. (**B**) Relative density of p-IκBα for IκBα. (**C**) Relative density of p-P65 for P65. Comparison with only LPS *p < 0.05; **p < 0.01; ***p < 0.001. Comparison with LCDE and LPS treated group ^##^*p* < 0.01; ^###^*p* < 0.001.

We performed western blotting to look at the effect of LCDE on the NF-κB pathway in LPS-stimulated HaCaT cells. The phosphorylation and degradation of IκBα are required steps in the activation of NF-κB. LCDE treatments significantly reduced LPS-induced IκBα and P65 degradation (Fig. 6). LCDE treatments reduced the expression of p-IκBα and p-P65 in a dose-dependent manner. These data suggest that LCDE has anti-inflammatory properties because it inhibits NF-κB activation in LPS-induced HaCaT cells.

### Molecular docking analysis with NF-κB and selected phenolic compounds

As shown in Table 2, the phenolic compounds sweroside, isoliquiritin, cardamonin, riboflavin, and arctigenin are thought to have a high peak area change rate to demonstrate potential antioxidant properties using DPPH binding HPLC. In addition, these compounds are included in LCDE, which is effective for anti-inflammation, using NF-κB, a typical inflammatory factor, molecular docking was used to confirm the difference in binding affinity.

**Figure 7:**
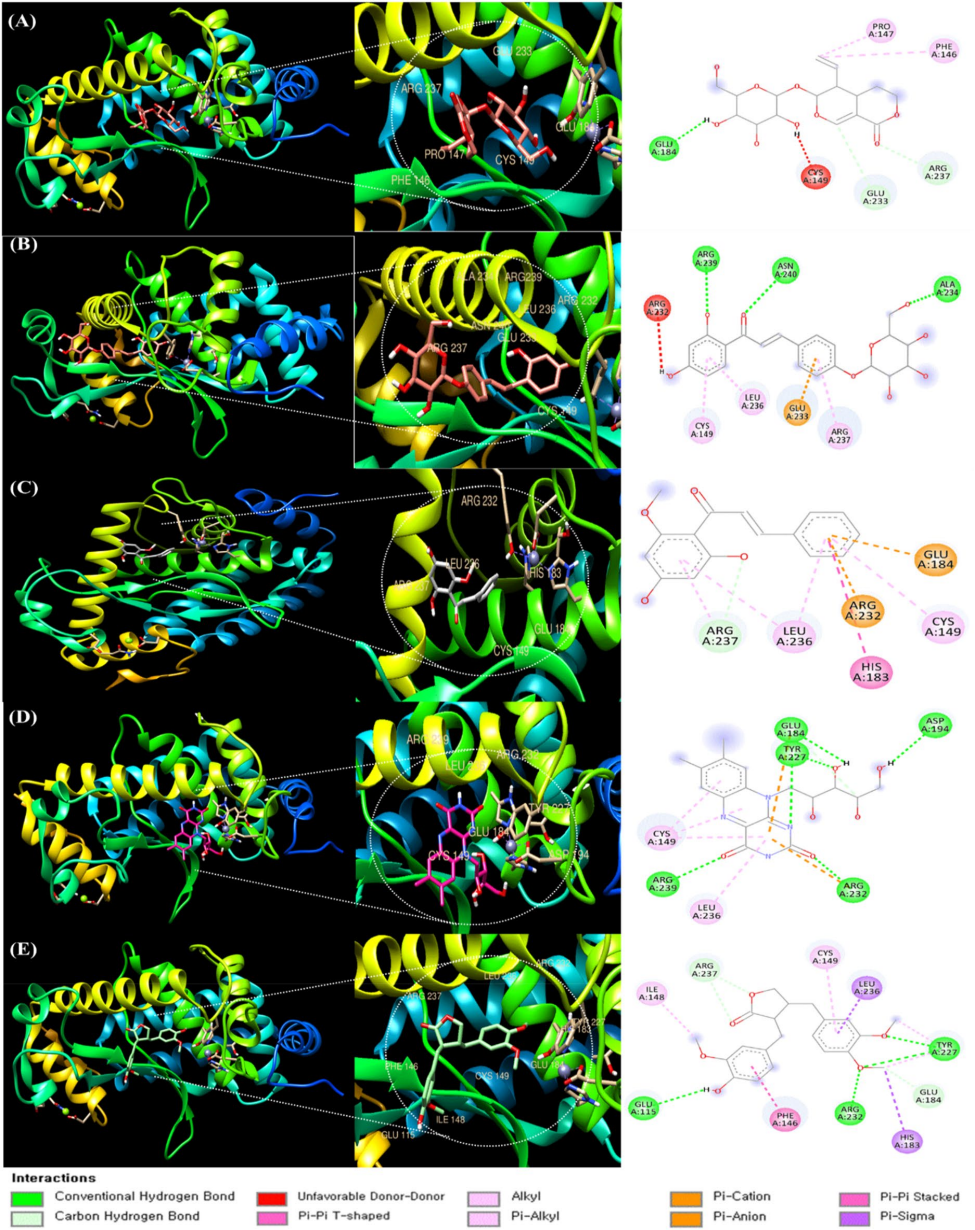
Molecular docking of phenolic compounds and NF-кB in LCDE. The 3D structure of NF-кB bound efficiently with (**A**) sweroside, (**B**) isoliquiritin, (**C**) cardamonin, (**D**) riboflavin, and (**E**) arctigenin.

The ligand–protein docking was analyzed using the UCSF Chimera program. Figure 7A shows active sites by sweroside and NF-кB. Additionally, several active sites (ARG237, CYS149, GLU187, GLU233, PHE146, PRO147) have been demonstrated to facilitate ligand binding. In Table 3, the molecular binding energy score was found to be − 6.6 kcal/mol.

**Table 3:** Molecular docking studies of LCDE phenolic compounds with NF-кB complex and their binding energy.

Binding ligand	Amino acid residue interactions	Binding affinity score
Sweroside	ARG237, CYS149, GLU187, GLU233, PHE146, PRO147	− 6.6 kcal/mol
Isoliquiritin	ARG,232, ARG237, ARG239, ALA234, ASN240, CYS149, GLU233, LEU236	− 6.0 kcal/mol
Cardamonin	ARG232, ARG237, CYS149, GLU184, HIS183, LEU236	− 6.7 kcal/mol
Riboflavin	ARG232, ARG239, ASP194, CYS149, GLU184, LEU236, TYR227	− 6.9 kcal/mol
Arctigenin	ARG232, ARG237, CYS149, GLU115, GLU184, HIS193, ILE148, LEU236, PHE146, TYR227	− 7.9 kcal/mol

In Fig. 7B, the active sites by isoliquiritin and NF-кB and several active sites (ARG,232, ARG237, ARG239, ALA234, ASN240, CYS149, GLU233, LEU236) have been demonstrated to promote ligand binding. The molecular binding energy score was − 6.0 kcal/mol in Table 3.

In Fig. 7C, the docking active sites (ARG232, ARG237, CYS149, GLU184, HIS183, LEU236) with cardamonin and NF-кB have been showed to promote ligand binding. The molecular binding energy score was found to be − 6.7 kcal/mol in Table 3.

Figure 7D shows that the active sites and active sites (ARG232, ARG239, ASP194, CYS149, GLU184, LEU236, TYR227) by riboflavin and NF-κB enable ligand binding. The molecular binding energy score was found to be − 6.9 kcal/mol in Table 3.

Figure 7E demonstrates how the docking and active sites (ARG232, ARG237, CYS149, GLU115, GLU184, HIS193, ILE148, LEU236, PHE146, TYR227) of arctigenin and NF-кB promote ligand binding. The molecular binding energy score was found to be − 7.9 kcal/mol in Table 3.

Looking at the docking results of five selected phenolic compounds and NF-кB with arctigenin had the highest relative binding affinity score and the binding sites were diverse. On the other hand, isoliquiritin had a relatively low binding affinity score of − 6.0 kcal/mol, but interacted with various binding sites.

## Discussion

**Figure 8:**
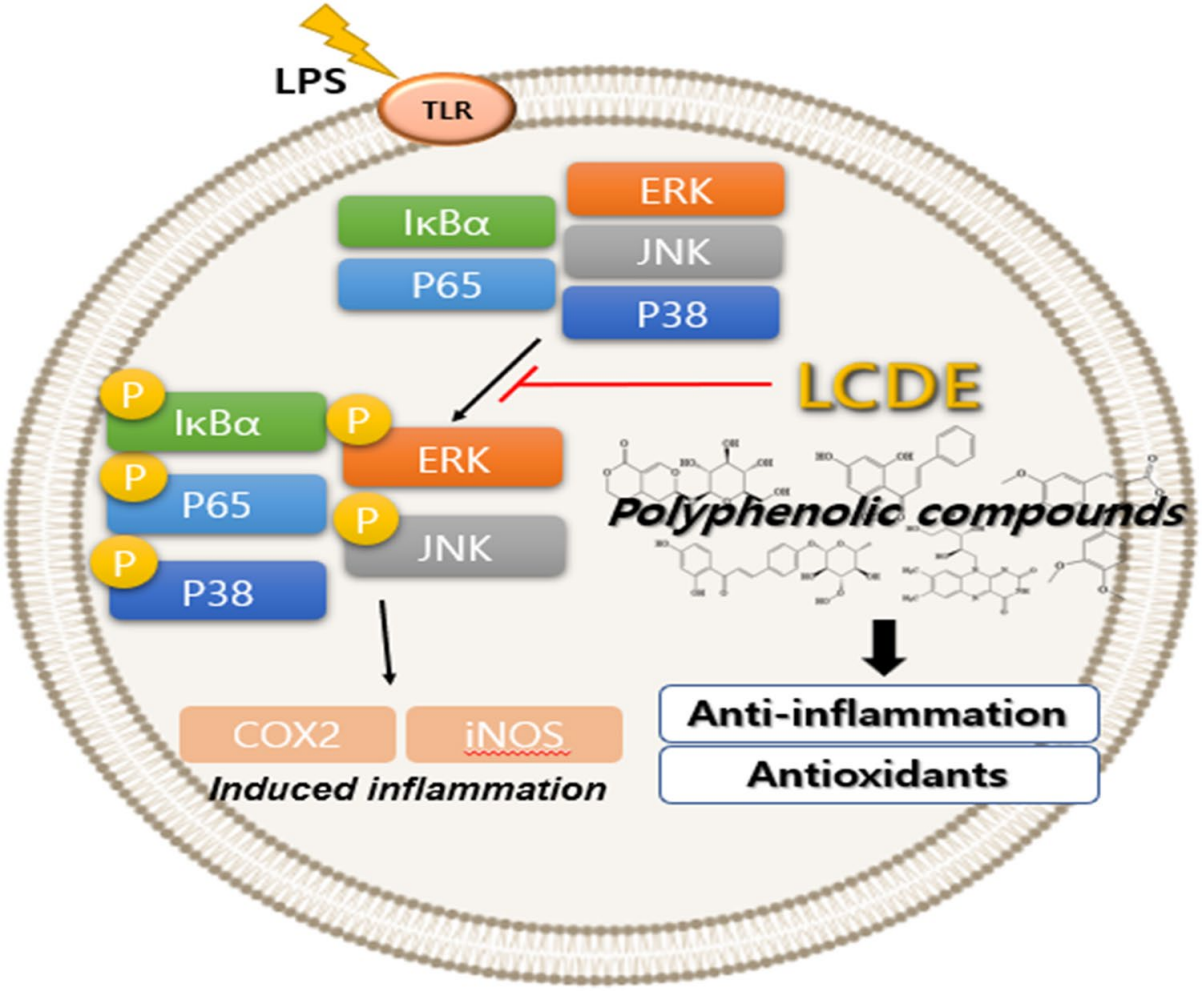
Anti-inflammatory effects of LCDE in HaCaT cells.

The phenolic compounds of the LCDE were identified in this work, and a high potential for antioxidant activity was found using a combined analysis of DPPH and HPLC. Following antioxidant studies, additional anti-inflammatory studies supported the finding that inflammation-induced keratinocytes showed suppressed phosphorylation of COX2 and iNOS, two representative inflammatory factors, and phosphorylation of MAPK and NF-кB pathway-related factors (Fig. 8).

Additionally, it was established that the phenolic compounds of LCDE had a noticeably high binding score in terms of structural binding through molecular docking of representative inflammation-related receptors NF-кB.

As a result, our work is the first to identify LCDE substances and screen DPPH ligand using UF-LC–MS analysis, based on the idea that unique organic chemicals and enzymes bind to target receptors^25^. These findings point to a way for screening complex compounds simultaneously in the early phases of drug development, as well as a strong tool for identifying possible oxidative stress and inflammatory ligands from a variety of medicinal plants.

However, research that links complex plant chemicals to biological targets or activities are difficult to conduct, and studies that provide experimental proof of components and targets are also missing^26^. Therefore, these results are good research data for research, and the extract and its components can be effective inhibitors of skin inflammation and act as antioxidants.

LCDE is a composite extract mixed with various medicinal substances extracted through distillation again. Even though it was a complex distillation extract called LCDE using the distillation method, phenolic components were identified, and antioxidant and anti-inflammatory effects were observed. Among the phenolic compounds identified in this distillation extract, the selected five compounds were sweroside, isoliquiritin, cardamonin, riboflavin and arctigenin. Sweroside regulates oxidative stress by inhibiting Keap1 and promoting Nrf2 signaling pathway in the pathway through the activation of Nrf2 (Nuclear factor E2-related factor 2) and Keap1 (Kelch-like ECH-associated protein) through ROS^27^. It also has a mechanism of reducing inflammatory cytokines and inhibiting the NF-κB signaling pathway^28^. Isoliquiritin contains antioxidant and anti-inflammatory properties and is used in the prevention and treatment of a variety of deseases^29^. Cardamonin also has antioxidant and anti-inflammatory effects, is involved in various pathways such as Nrf2, ERK and mTOR, and downregulates COX2 and iNOS through inhibition of NF-κB^30,31^. Riboflavin is also known as vitamin B2, and when oxidative stress increases, riboflavin intake activates antioxidant enzymes^32^. Arctigenin has an anti-inflammatory effect through iNOS inhibition due to inactivation of the JAK-STAT pathway, and the anti-inflammatory effect caused by the decrease in iNOS is related to the antioxidant effect, suggesting that it has an antioxidant effect by reducing ROS production^33^.

All of these five phenolic compounds have antioxidant and anti-inflammatory effects, and are noteworthy in that they exist and show effects even after extraction through pure distillation after plant extraction.

In molecular docking assay, one thing to note about results is to keep in mind that the outcomes of the molecular docking scores are not examined by determining the precise binding affinity. Even when high results are reached, there are situations where the interaction structures do not align properly, in which case absolute docking results are useless and need to a procedure to visually check the structural binding is essential.

Looking at Table 3, arctigenin showed the highest relative binding affinity score. On the other hand, isoriquiritin had a relatively low binding affinity score, but interacted with various binding sites. Therefore, it is important to note that all five candidate phenolic compounds selected for docking with the inflammatory factor NF-кB are proposed as excellent anti-inflammatory candidates.

In this result, the greater the change in relative area value among the phenolic components of the LCDE selected through HPLC–MS/MS binding with DPPH, that is, the higher the docking affinity score of the component considered to have a higher antioxidant effect. This is because the antioxidant and anti-inflammatory effects of the phenolic component of the selected LCDE are closely related^34^ and it is expected that the selected ingredients will be widely used in antioxidant and anti-inflammatory research in the future.

Although many studies on the anti-inflammatory and antioxidant effects of various herbal preparations have already been conducted by distillation extraction^35^, there are not many contents through isolation and identification of the components as in this study and molecular docking binding ability. Therefore, it is expected that this method will be widely used.

## Material and methods

### Plant material

The *Lonicera japonica & Chenpi* distillation extract (LCDE) was made by mixing *Lonicera japonica, Taraxacum* (Code Number: 00225A)*, Chenpi* (00891A)*, Forsythia* (00232A), and *Licorice* (00258A). The plants used in this study were cultivated by farmers at a specialized farm in the Jirisan Mountain of Gyeongsangnam-do, and are assigned a code number after complete identification of the material by professional identification personnel at the Animal Bio Resource Bank (http://www.abrb.or.kr). Afterwards, it was stored in the herbarium for distribution and research purposes. The Animal Biologically Active Substances Resource Bank is a nationally designated research data bank that has optimal storage conditions for the storage and distribution of research materials. These plants were washed with water, then cut into small pieces, and dried in an oven (55 °C for 72 h). Until use, the mixture plants are stored in sealing polyethylene bags with silica gel at − 20 °C.

### Reagents and standards

The DPPH (2,2-Diphenyl-1-picrylhdrazyl) was provided by Sigma-Aldrich Corp (St. Louis, MO, USA. cas no. 1898-66-4). 3-(4,5-Dimethylthiazol-2-yl)-2,5-diphenyltetrazolium bromide (MTT) was purchased from Duchefa Biochemie (Haarlem, the Netherlands). Antibodies to COX-2 (cat. no. 12282S), iNOS (cat. no. 13120S), p65 (cat. no. 8242S), phosphorylated p65 (p-p65) (cat. no. 3033S), IкBα (cat. no. 4812S), phosphorylated IкBα (p-IкBα) (cat. no. 2859S), JNK (Jun N-terminal kinase) (cat. no. 9258S), phosphorylated JNK (p-JNK) (cat. no. 4671S), ERK (Extracellular-signal-regulated kinase) (cat. no. 4695S), phosphorylated ERK (p-ERK) (cat. no. 4370S), p38 (cat. no. 8690S), phosphorylated p38 (p-p38) (cat. no. 4511S), and β-actin (cat. no. 4970S) were purchased from Cell Signaling Technology (Danvers, MA, USA). Horseradish peroxidase (HRP)-conjugated secondary antibodies to antirabbit (cat. no. A120-101P) and antimouse (cat. no. A90-116P) were obtained from Bethyl Laboratories, Inc. (Montgomery, AL, USA).

### Extraction and purification of LCDE phenolic compounds

Boil the plants mixture of *Lonicera japonica* (15 g), *Taraxacum* (10 g), *Chenpi* (10 g), *Forsythia* (10 g) and *Licorice* (5 g) in 4L of water at 90 °C for two days. By heating distilled water, the active ingredients were evaporated together with the water and extracted. Filter paper Whatman qualitative No. 6 was used to separate the mixture. The mixture was concentrated to 500 mL at decreased pressure and 45 °C using a rotary evaporator (N-1110, Eyela, Tokyo, Japan) spinning at 100 revolutions per minute. To get rid of fatty particles, the concentrate was washed three times with 500 mL of hexane. With 250 mL of ethyl acetate, the residual filtrate was extracted three times. To get rid of the highly polar components, the residue was first dehydrated with MgSO4 and then eluted using silica gel solvent (40 cm 2.5 cm) and ethyl acetate. Under lower pressure, the solvent was condensed. Afterward, it was frozen dried to produce a mixed phenolic powder (0.2 g, 0.4% of raw dried plants). It was dissolved at 1000 ppm for further study and stored at − 70 °C.

### HPLC and LC–MS/MS analysis

HPLC and LC–MS/MS was performed on a 1260 series HPLC system (Aglient Technologies, Inc., California, USA) and ultra quadrupole time of flight LC–MS/MS X500R system (AB Scies, Framingham, MA) operated in positive ion mode. A gradient system with a flow rate of 0.5 mL/min was used for analysis, and a Prontosil C18 column (length, 250 mm; inner diameter, 4.6 mm; particle size, 5 m; Phenomenex Co., Ltd., California, USA; Biochoff Chromatography) was used. Acetonitrile and DW, both of which contained 0.1% formic acid, served as the solvent. The mobile phases were subjected to the following solvent conditions: 0–10 min at 10–15% B, 10–20 min at 20% B, 20–30 min at 25%, 30–40 min at 40%, 40–50 min at 70%, 50–60 min at 95%, and 60–70 min at 95%. The analysis was carried out at a temperature of 35 °C at a wavelength of 284 nm. Calculations of the peak areas obtained from UV and reference materials were utilized to determine the concentration of phenolic compounds.

### Antioxidant activity using DPPH binding HPLC technology

1000 ppm LCDE powder and 0.2 mg/mL DPPH reagents were mixed in a ratio of 6:1 (v:v) and reacted at room temperature for 15 min. Prior to HPLC analysis, the mixture was filtered through a 0.45 m filter, and methanol was employed as a control in place of the DPPH reagent. By looking at the chromatographic peak values and standard curve values of the samples and controls that underwent the DPPH reaction, it was possible to identify the composition of the chemical that reacted with it. This allows for the identification of the primary antioxidant elements in LCDE phenolic compounds.

### Cell culture and LCDE treatment

The HaCaT cells obtained from the thermo fisher Scientific were grown in full DMEM with 10% heat-inactivated fetal bovine serum (FBS) with 100 U/ml penicillin and 100 μg/ml streptomycin. The cells were incubated at 37 °C in a humidified environment with 5% CO_2_. After seeding the cells, At 37 °C, the cells were incubated in a humid environment with 5% CO2.

### Cell viability assay

HaCaT cells were seeded at a density of 1 × 10^4^cells per well in 96 well plates and then cultured, with LPS (1 µg/mL) and co-treatment with various concentrations of LCDE (0, 0.1, 0.25, 0.5, 0.75, 1, 1.25, 2.5, 5, 7.5 and 10 µg/mL) at 37 °C for 24 h. After incubation, MTT solution (10 µl; 5 mg/ml) was added to the plate and incubated at 37 °C for ~ 2 h. The insoluble formazan crystals were then dissolved in DMSO after the growth media was entirely washed away. And the absorbance of the converted dye was measured at a wavelength of 560 nm by microplate reader Multiskan FC (Thermo Scientific, Rockford, IL, USA).

### Western blot

For western blot analysis, HaCaT cells were seeded into 60 mm plates at a density of 1 × 10^6^ cells/well and treated with 0.1 and 0.25 µg/mL LCDE for 24 h at 37 °C. Then the cells were lysed in ice-cold RIPA buffer (50 mM Tris–HCL (pH 8.0), 0.5% sodium deoxycholate, 1 mM EDTA, 150 mM NaCl, 0.1 SDS and 1% NP-40). Protein concentrations were determined using the Pierce™ BCA Protein Assay (Thermo Scientific, Rockford, IL, USA) according to the manufacturer’s instructions. Equal amounts of protein (10 μg) were separated via SDS-PAGE on 10% gels and transferred onto PVDF membranes using the JP/WSE-4040 HorizeBLOT 4 M-R WSE-4045 (ATTO Blotting System, USA). The blots were then blocked with EzBlockChemi (ATTO Blotting System, Japan) for 1 h at room temperature. Membranes were further incubated with 1:1000 dilutions of primary antibodies overnight at 4 °C. The membranes were washed three times for 10 min with TBS-T and probed with a second antibody until 2 h at room temperature. The second antibody was diluted at 1:5000. The blots were visualized using Clarity™ ECL substrate reagent (Bio Rad Laboratories, Inc.) and quantified by densitometry using Image J software (National Institutes of Health) with β-actin as the loading control. The experiment was performed in triplicate.

### Molecular docking

The structure of NF-κB was obtained at high resolution from a protein data bank (PDB) (https://www.rcsb.org/, accessed on 28 April 2022) with PDB ID 4Q3J (NF-κB), and the three-dimensional structures of the selected phenolic compound of LCDE were obtained from PubChem (https://pubchem.ncbi.nlm.nih.gov/, accessed on 28 April 2023) with PDB sweroside (Compound CID: 161036), isoliquiritin (Compound CID: 5318591), cardamonin (Compound CID: 641785), riboflavin (Compound CID: 493570), and arctigenin (Compound CID: 28125531). The protein and ligand were docked using the USCF Chimera tool, and all possible conformations were returned using default parameters. The results were visualized by PyMOL and Discovery Studio (DeLano, 2002). The estimated free energy of binding and total intermolecular energy was used to evaluate the results.

### Statistical analysis

The test measurements were expressed as mean ± standard deviation (M ± SD) in triplicate measurements. Statistical analysis was performed using spss version 12.0 (SPSS Inc, m Chicago, IL, USA), and one-way factorial analysis of variance (ANOVA). Statistical significance was analyzed by Duncan’s multiple range and Student’s test at p < 0.05 level, after one-way analysis of variance. (# *p* < 0.05, ## *p* < 0.01, ### *p* < 0.001 vs. untreated, positive control group; and * *p* < 0.05, ** *p* < 0.01, *** *p* < 0.001 vs. LPS-treated, negative control group).

## Conclusion

Through this study, phenolic compounds were identified in LCDE, a complex distillation extract, and the antioxidant effect of each selected component was confirmed, and the anti-inflammatory effect of LCDE was observed by inducing inflammation in the target skin keratinocytes. In addition, the binding affinity of the selected phenolic compound with the inflammation-related protein was confirmed through molecular docking. Depending on the phenolic compound composition of these complex distillate extracts, LCDE could be a potential drug for various inflammation-related pathways, structural binding affinity and antioxidant effects.

### Supplementary Information

Supplementary Information.

## Data Availability

The data used to support the findings of this study are available upon request from the corresponding author.

## References

[1] Ji Hyun B, Mi Soon K, Eun Hae K (2005). Antimicrobial effect of *Lonicerae flos* extracts on food-borne pathogens. Korean J. Food Sci. Technol..

[2] Heo S-I, Wang M-H (2008). Antioxidant activity and cytotoxicity effect of extracts from *Taraxacum mongolicum* H. Korean J. Pharmacogn..

[3] Sung-Gu L, Sung-Cheon O, Jae-Seon J (2015). Antioxidant activities of citrus unshiu extracts obtained from different solvents. Korean J. Food Nutr..

[4] Mi Jin K (2006). Skin anti-aging effect of *Forsythia viridissima* L. Extract. KSBB J..

[5] Sung K-C (2006). A study on the pharmacetical characteristics & analysis of glycyrrhizin extract. 한국유화학회지.

[6] Li Q (2017). The anti-inflammatory effect of Sonchus oleraceus aqueous extract on lipopolysaccharide stimulated RAW 264.7 cells and mice. Pharm. Biol..

[7] Torres-Rodriguez ML (2016). Anti-inflammatory and antioxidant effect of *Calea urticifolia* lyophilized aqueous extract on lipopolysaccharide-stimulated RAW 2647 macrophages. J. Ethnopharmacol..

[8] Kim YJ (2018). Anti-inflammatory effects of *Angelica sinensis* (Oliv.) Diels water extract on RAW 264.7 induced with lipopolysaccharide. Nutrients.

[9] Yoon SB (2009). Anti-inflammatory effects of *Scutellaria baicalensis* water extract on LPS-activated RAW 264.7 macrophages. J. Ethnopharmacol..

[10] Li XJ (2015). *Gynura procumbens* reverses acute and chronic ethanol-induced liver steatosis through MAPK/SREBP-1c-dependent and -independent pathways. J. Agric. Food Chem..

[11] Poh T-F (2013). *Gynura procumbens* causes vasodilation by inhibiting angiotensin II and enhancing bradykinin actions. J. Cardiovasc. Pharmacol..

[12] Chen GL (2019). Antioxidant and anti-inflammatory properties of flavonoids from lotus plumule. Food Chem..

[13] Kim H, Han TH, Lee SG (2009). Anti-inflammatory activity of a water extract of *Acorus calamus* L. leaves on keratinocyte HaCaT cells. J. Ethnopharmacol..

[14] Chan CX, Zug KA (2021). Diagnosis and management of dermatitis, including atopic, contact, and hand eczemas. Med. Clin. N. Am..

[15] Clark GW, Pope SM, Jaboori KA (2015). Diagnosis and treatment of seborrheic dermatitis. Am. Fam. Physician.

[16] Woo YK (2020). The anti-inflammatory and anti-apoptotic effects of advanced anti-inflammation composition (AAIC) in heat shock-induced human HaCaT keratinocytes. J. Cosmet. Dermatol..

[17] Yang CY (2022). Antioxidant, anti-inflammation and antiaging activities of *Artocarpus altilis* methanolic extract on urban particulate matter-induced HaCaT keratinocytes damage. Antioxidants (Basel).

[18] Okayama Y (2005). Oxidative stress in allergic and inflammatory skin diseases. Curr. Drug Targets Inflamm. Allergy.

[19] Han H (2014). Characterization of metabolites of sweroside in rat urine using ultra-high-performance liquid chromatography combined with electrospray ionization quadrupole time-of-flight tandem mass spectrometry and NMR spectroscopy. J. Mass Spectrom..

[20] Guo Y (2022). Content determination and release characteristics of six components in the different phases of "Glycyrrhizaglabra-Nux vomica" decoction by UPLC-MS/MS. Molecules.

[21] Dong F (2020). Systematic screening and characterization of cardamonin metabolites using UHPLC-Q-Exactive Orbitrap MS after oral administration to rats. Arab. J. Chem..

[22] Insińska-Rak M (2020). Riboflavin degradation products; combined photochemical and mass spectrometry approach. J. Photochem. Photobiol. A Chem..

[23] Zou Q (2013). Development of an LC/MS/MS method in order to determine arctigenin in rat plasma: its application to a pharmacokinetic study. Biomed. Chromatogr..

[24] Alhallaf W, Perkins LB (2022). The anti-inflammatory properties of chaga extracts obtained by different extraction methods against LPS-induced RAW 264.7. Molecules.

[25] Kim HH (2021). Potential antioxidant and anti-inflammatory function of *Gynura procumbens* polyphenols ligand. Int. J. Mol. Sci..

[26] Zhu H (2013). Bioactivity fingerprint analysis of cyclooxygenase-2 ligands from radix Aconiti by ultrafiltration-UPLC-MSn. Anal. Bioanal. Chem..

[27] Li J (2021). Sweroside protects against myocardial ischemia-reperfusion injury by inhibiting oxidative stress and pyroptosis partially via modulation of the Keap1/Nrf2 Axis. Front. Cardiovasc. Med..

[28] Wang J (2021). Anti-inflammatory effects of sweroside on LPS-induced ALI in mice via activating SIRT1. Inflammation.

[29] Peng F (2015). A review: The pharmacology of isoliquiritigenin. Phytother. Res..

[30] Peng YJ (2021). Cardamonin attenuates inflammation and oxidative stress in interleukin-1beta-stimulated osteoarthritis chondrocyte through the Nrf2 pathway. Antioxidants (Basel).

[31] Goncalves LM, Valente IM, Rodrigues JA (2014). An overview on cardamonin. J. Med. Food.

[32] Olfat N, Ashoori M, Saedisomeolia A (2022). Riboflavin is an antioxidant: A review update. Br. J. Nutr..

[33] Kou X (2011). Arctigenin inhibits lipopolysaccharide-induced iNOS expression in RAW264.7 cells through suppressing JAK-STAT signal pathway. Int. Immunopharmacol..

[34] Arulselvan P (2016). Role of antioxidants and natural products in inflammation. Oxid. Med. Cell Longev..

[35] Nirmal SA (2012). Analgesic and anti-inflammatory activity of beta-sitosterol isolated from *Nyctanthes arbortristis* leaves. Inflammopharmacology.

