# Author Correction: Antioxidant effects of phenolic compounds in through the distillation of *Lonicera japonica & Chenpi* extract and anti-inflammation on skin keratinocyte

**DOI:** 10.1038/s41598-023-50042-2

**Published:** 2023-12-20

**Authors:** Hun Hwan Kim, Se Hyo Jeong, Min Yeong Park, Pritam Bhangwan Bhosale, Abuyaseer Abusaliya, Hyun Wook Kim, Je Kyung Seong, Meejung Ahn, Kwang Il Park, Gon Sup Kim

**Affiliations:** 1 Research Institute of Life Science and College of Veterinary Medicine, Gyeongsang National University, Jinju, 52828 Republic of Korea; 2 Division of Animal Bioscience & Intergrated Biotechnology, Jinju, 52725 Republic of Korea; 3 Laboratory of Developmental Biology and Genomics, College of Veterinary Medicine, BK21 PLUS Program for Creative Veterinary Science Research, Research Institute for Veterinary Science, Seoul National University, Seoul, 08826 Republic of Korea; 4 Department of Animal Science, College of Life Science, Sangji University, Wonju, 26339 Republic of Korea

Correction to: *Scientific Reports* 10.1038/s41598-023-48170-w, published online 28 November 2023

The original version of the Article contained an error in the Author Information section, where the statement of equal contribution was implemented erroneously: “These authors contributed equally: Hun Hwan Kim and Se Hyo Jeong.” Hun Hwan Kim is the first author and Se Hyo Jeong is the second author of this Article.

The original Article has been corrected.

