# Author Correction: Antioxidant effects of phenolic compounds in through the distillation of *Lonicera japonica & Chenpi* extract and anti-inflammation on skin keratinocyte

**DOI:** 10.1038/s41598-024-53473-7

**Published:** 2024-02-05

**Authors:** Hun Hwan Kim, Se Hyo Jeong, Min Yeong Park, Pritam Bhangwan Bhosale, Abuyaseer Abusaliya, Hyun Wook Kim, Je Kyung Seong, Meejung Ahn, Kwang Il Park, Gon Sup Kim

**Affiliations:** 1 Research Institute of Life Science and College of Veterinary Medicine, Gyeongsang National University, Jinju, 52828 Republic of Korea; 2 Division of Animal Bioscience & Intergrated Biotechnology, Jinju, 52725 Republic of Korea; 3 Laboratory of Developmental Biology and Genomics, BK21 PLUS Program for Creative Veterinary Science Research, Research Institute for Veterinary Science, College of Veterinary Medicine, Seoul National University, Seoul, 08826 Republic of Korea; 4 Department of Animal Science, College of Life Science, Sangji University, Wonju, 26339 Republic of Korea

Correction to: *Scientific Reports* 10.1038/s41598-023-48170-w, published online 28 November 2023

The original version of this Article contained an error in the Material and methods section, under the subheading ‘Cell culture and LCDE treatment’, where an instance of the unit ‘μg/ml’ was incorrectly stated as “g/ml”.

“The HaCaT cells obtained from the thermo fisher Scientific were grown in full DMEM with 10% heat-inactivated fetal bovine serum (FBS) with 100 U/ml penicillin and 100 g/ml streptomycin.”

now reads:

“The HaCaT cells obtained from the thermo fisher Scientific were grown in full DMEM with 10% heat-inactivated fetal bovine serum (FBS) with 100 U/ml penicillin and 100 μg/ml streptomycin.”

The original Article has been corrected.

