# Publisher Correction: Antioxidant effects of phenolic compounds in through the distillation of *Lonicera japonica* & *Chenpi* extract and anti-inflammation on skin keratinocyte

**DOI:** 10.1038/s41598-024-64515-5

**Published:** 2024-06-17

**Authors:** Hun Hwan Kim, Se Hyo Jeong, Min Yeong Park, Pritam Bhangwan Bhosale, Abuyaseer Abusaliya, Hyun Wook Kim, Je Kyung Seong, Meejung Ahn, Kwang Il Park, Gon Sup Kim

**Affiliations:** 1 Research Institute of Life Science and College of Veterinary Medicine, Gyeongsang National University, Jinju, 52828 Republic of Korea; 2 Division of Animal Bioscience & Intergrated Biotechnology, Jinju, 52725 Republic of Korea; 3 Laboratory of Developmental Biology and Genomics, BK21 PLUS Program for Creative Veterinary Science Research, Research Institute for Veterinary Science, College of Veterinary Medicine, Seoul National University, Seoul, 08826 Republic of Korea; 4 Department of Animal Science, College of Life Science, Sangji University, Wonju, 26339 Republic of Korea

Correction to: *Scientific Reports* 10.1038/s41598-023-48170-w, published online 28 November 2023

The original version of this Article contained an error in Figure 4, 5 and 6, where the symbol “μ” was not displayed correctly in panel (A). The original Figure 4, 5 and 6 and accompanying legends appear below.

**Figure 4:**
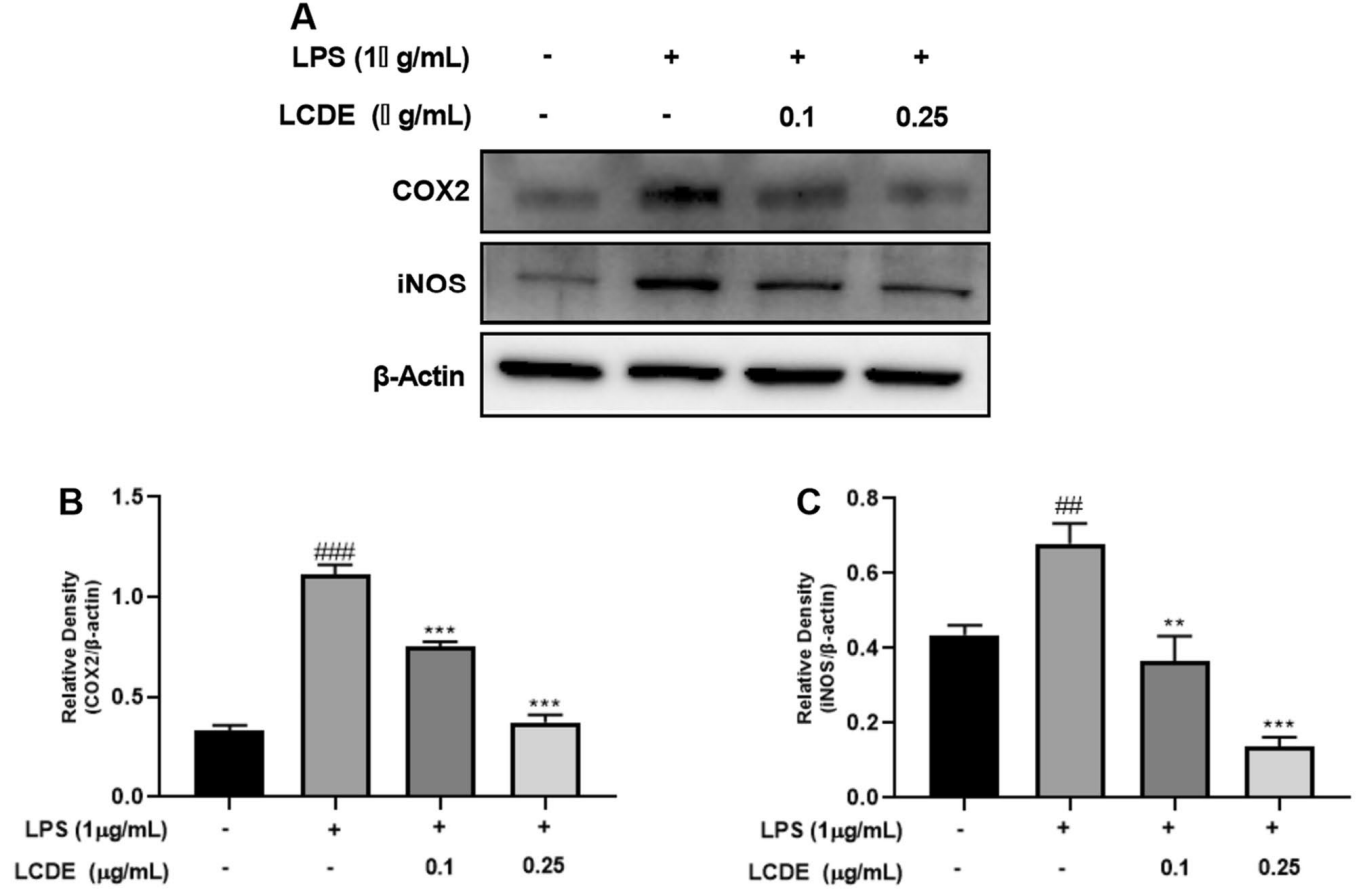
Effect of LCDE on COX-2 and iNOS expression in with or without LPS-stimulated HaCaT cells. HaCaT cells were treated with LCDE (0, 0.1 and 0.25 μg/mL) with or without LPS (1 μg/mL) for at 37 °C 24 h. (**A**) The expression of COX2 and iNOS was quantified by western blot analysis. (**B**) Relative density of COX-2 expression. (**C**) Relative density of iNOS expression. Comparison with LCDE and LPS treated group **p < 0.01; ***p < 0.001. Comparison with LPS treated group ^##^*p* < 0.01; ^###^*p* < 0.001.

**Figure 5:**
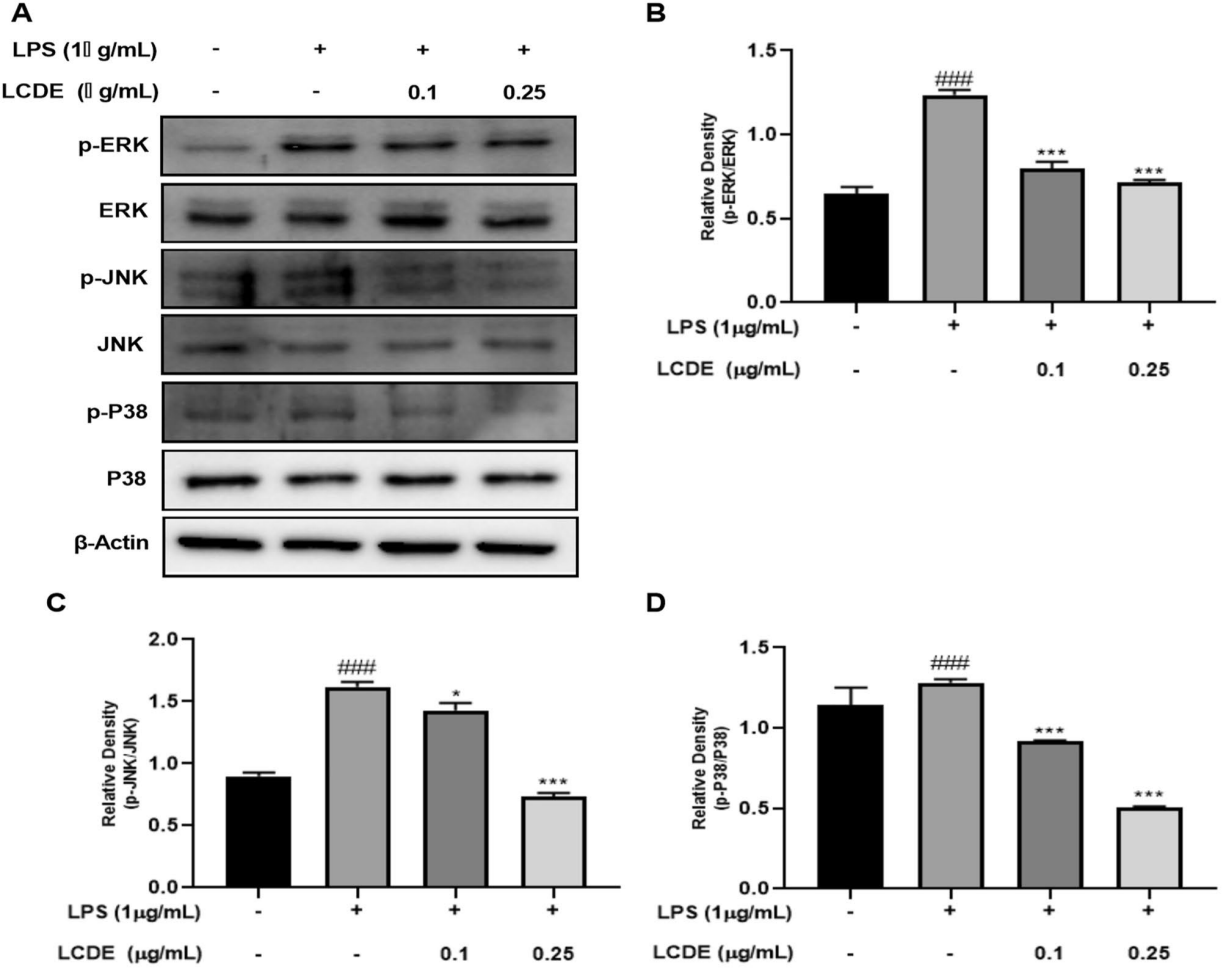
Effect of LCDE on with or without LPS induced MAPKs protein expression in HaCaT cells. HaCaT cells were treated with LCDE (0, 0.1 and 0.25 μg/mL) with or without LPS (1 μg/mL) for at 37 °C 24 h. (**A**) The expression of MAPKs was quantified by western blot analysis. (**B**) Expression of p-ERK affected by LCDE. (**C**) Expression of p-JNK affected by LCDE. (**D**) Expression of p-P38 affected by LCDE. Comparison with only LPS *p < 0.05; ***p < 0.001. Comparison with LCDE and LPS treated group ^##^*p* < 0.01; ^###^*p* < 0.001.

**Figure 6:**
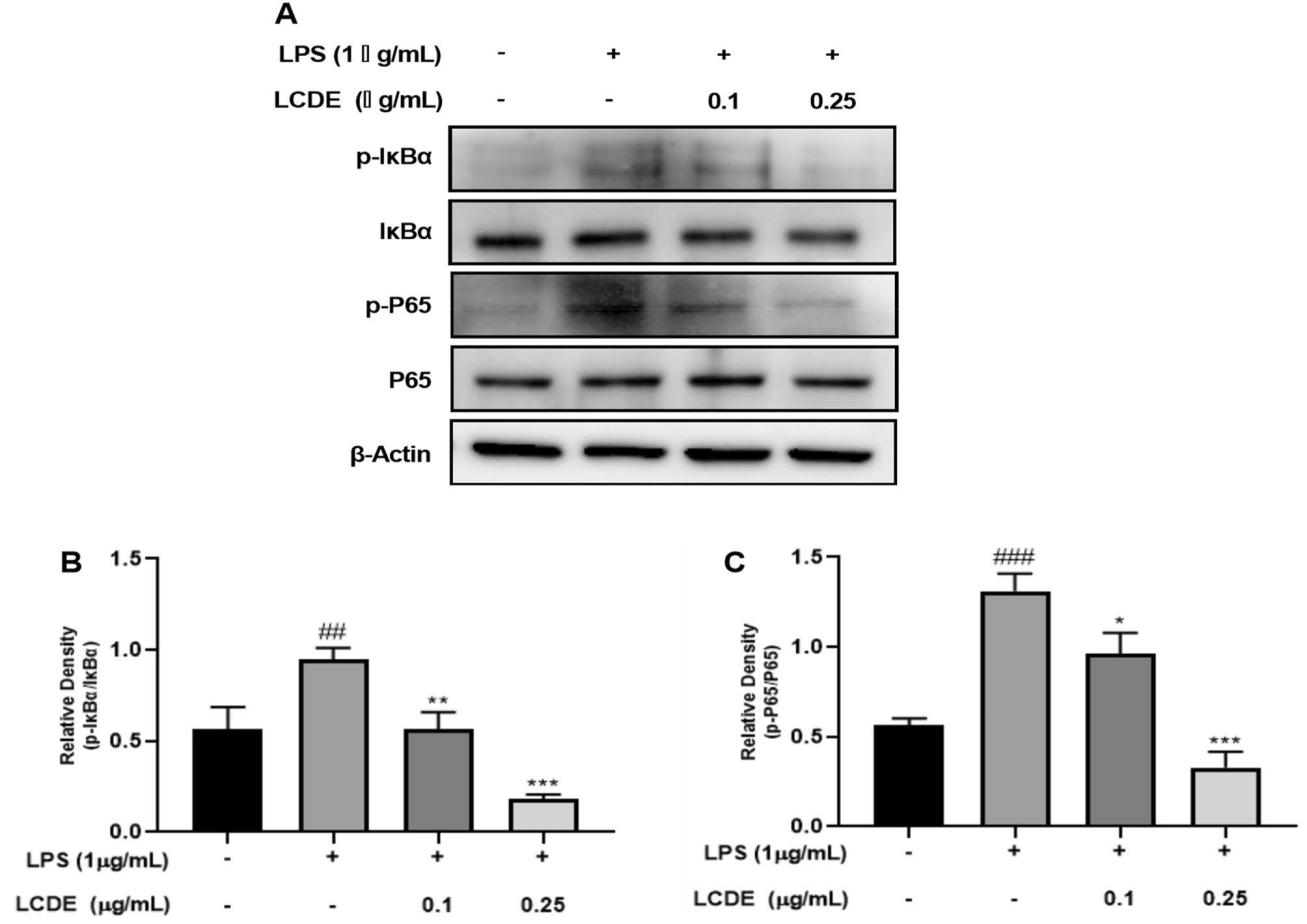
Effect of LCDE on with or without LPS induced protein expression of NF-κB in HaCaT cells. HaCaT cells were treated with LCDE (0, 0.1 and 0.25 μg/mL) with or without LPS (1 μg/mL) for at 37 °C 24 h. (**A**) The expression of NF-κB were quantified by western blot analysis. (**B**) Relative density of p-IκBα for IκBα. (**C**) Relative density of p-P65 for P65. Comparison with only LPS *p < 0.05; **p < 0.01; ***p < 0.001. Comparison with LCDE and LPS treated group ^##^*p* < 0.01; ^###^*p* < 0.001.

The original Article has been corrected.

