# Supplementary figures and images for: Antioxidant effects of phenolic compounds in through the distillation of Lonicera japonica & Chenpi extract and anti-inflammation on skin keratinocyte
Source: Sci Rep. 2023 Nov 28;13:20883. doi: 10.1038/s41598-023-48170-w (PMC10684860; doi:10.1038/s41598-023-48170-w)

# LCDE FULL BAND

$\beta$ -actin

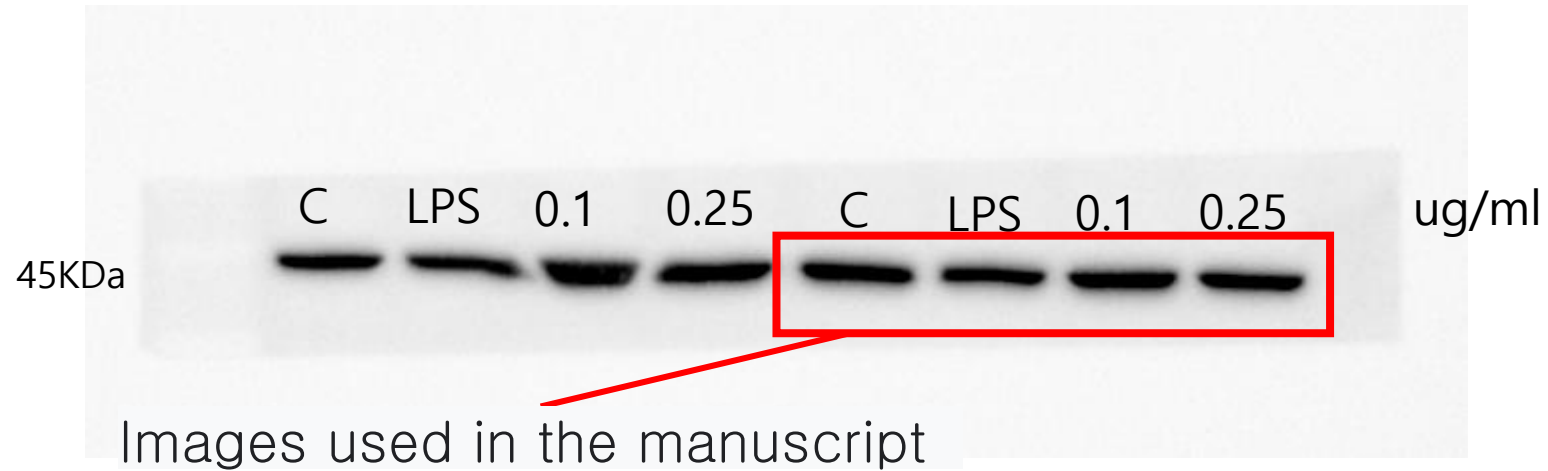

COX-2

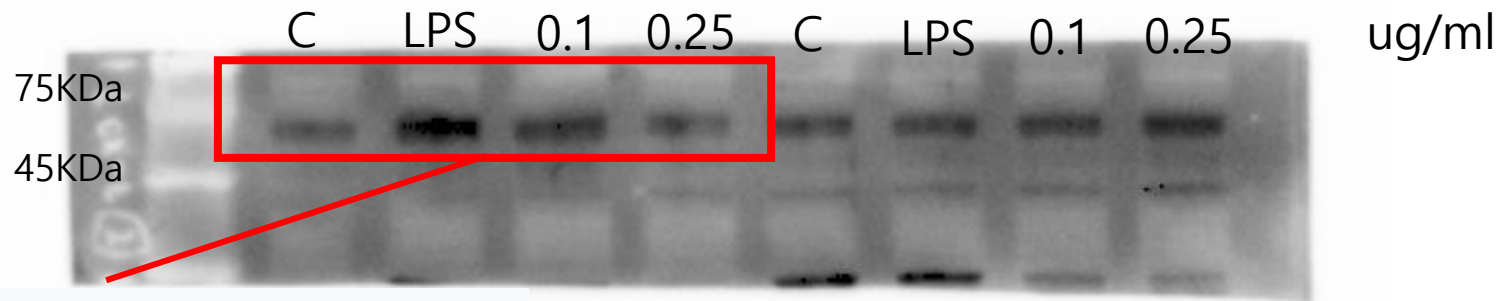

INOS

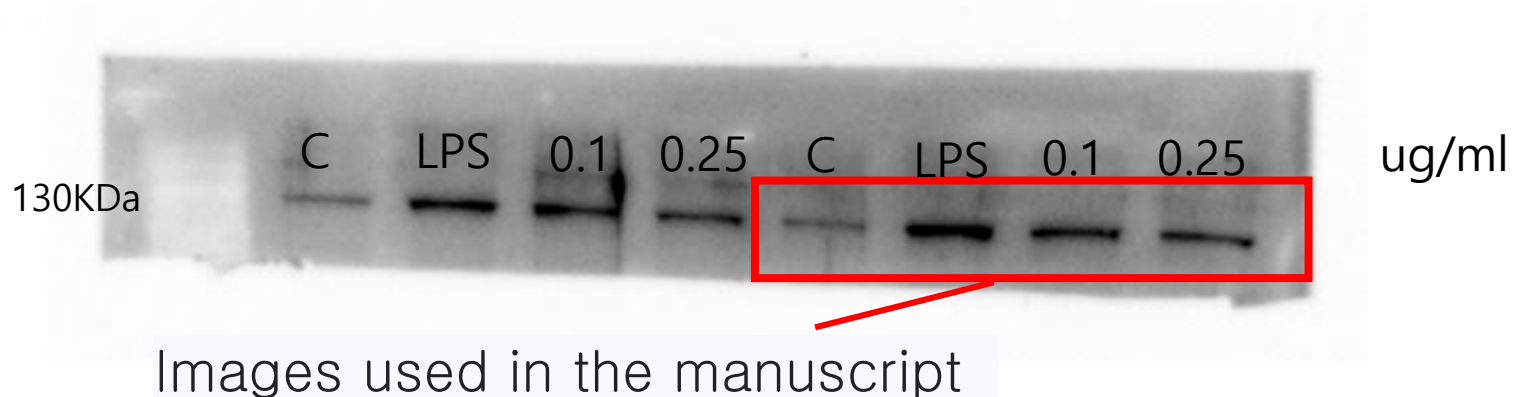

p-ERK

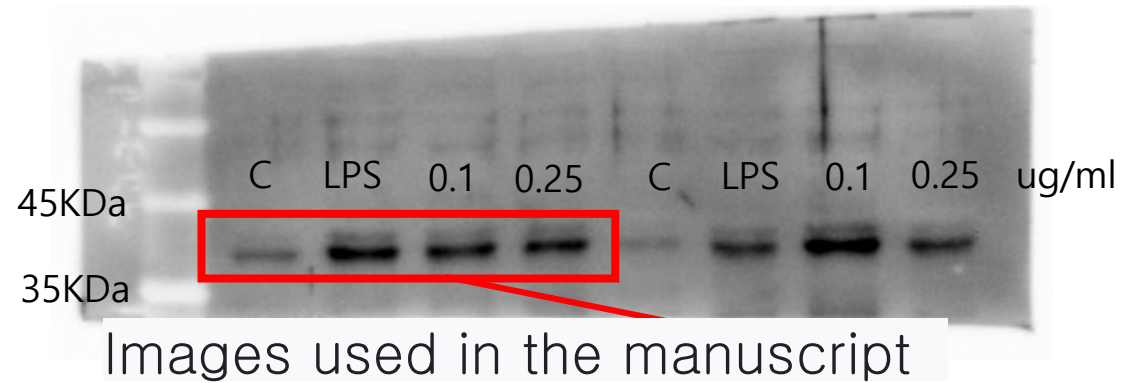

ERK

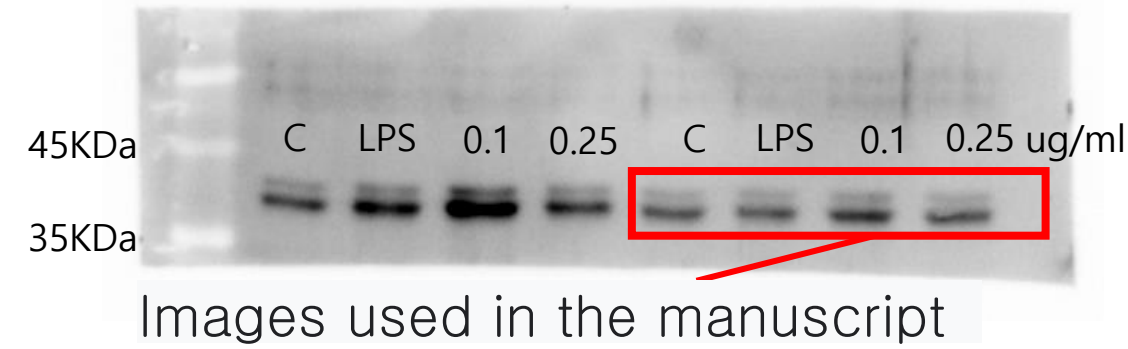

p-JNK

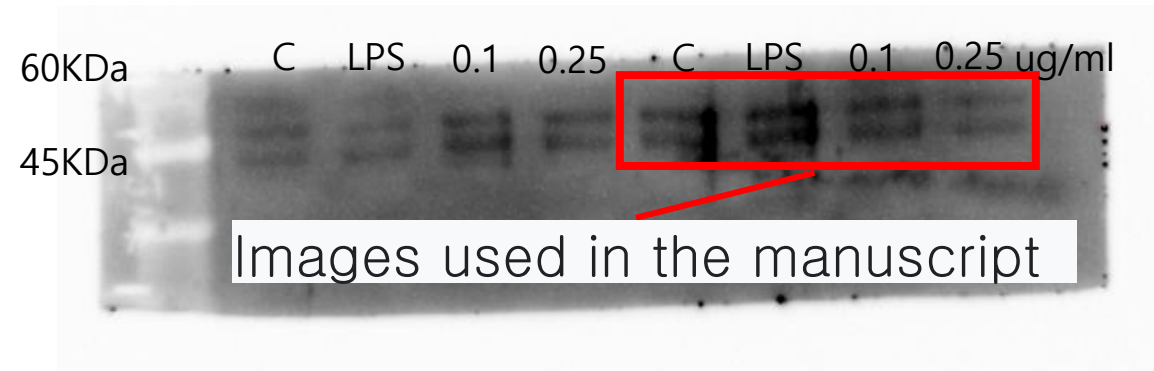

JNK

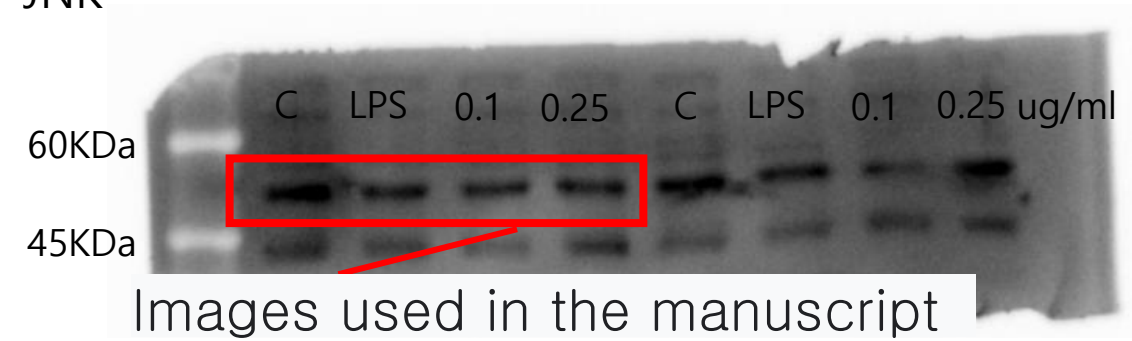

p-P38

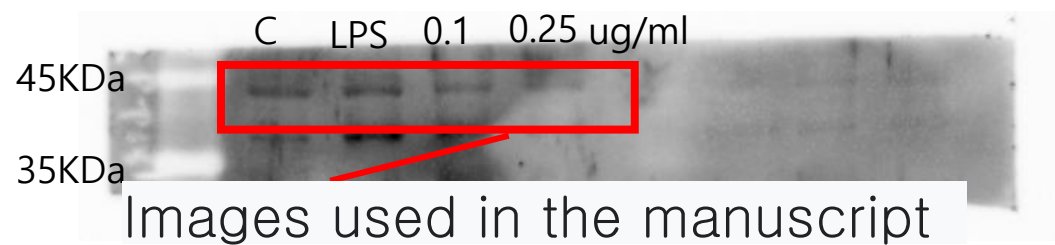

P38

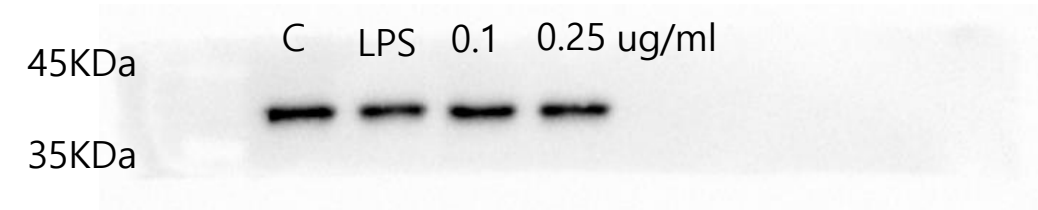

p-IkBa

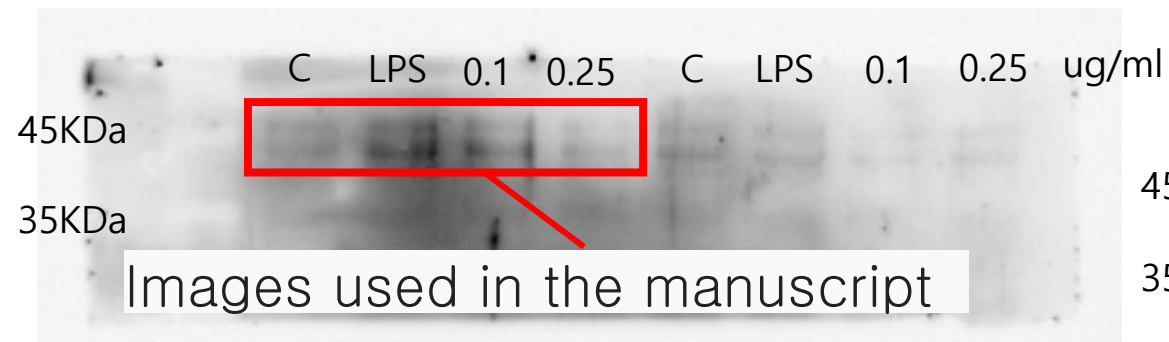

IkBa

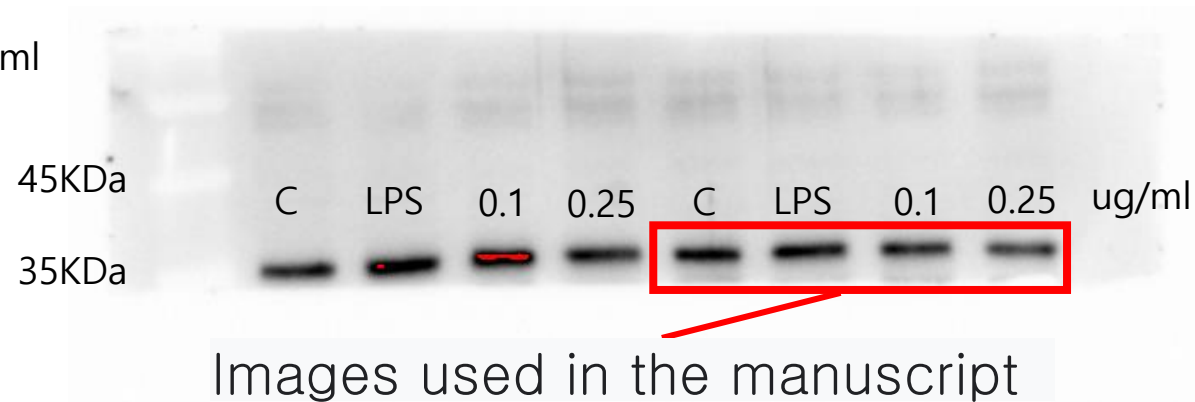

p-P65

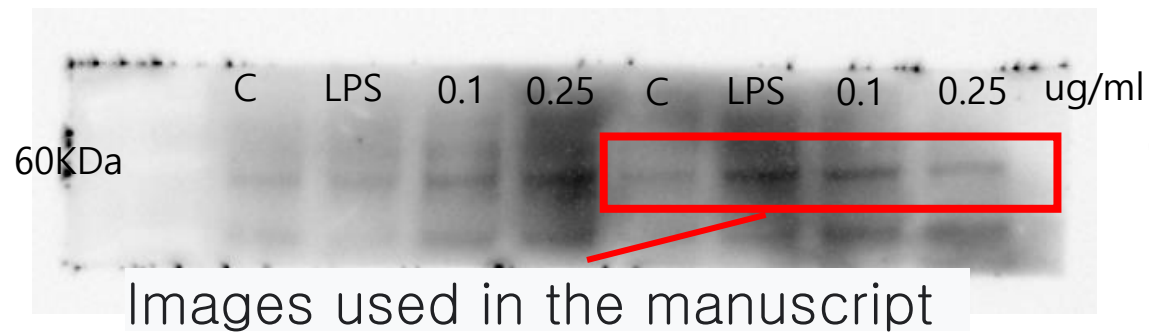

P65

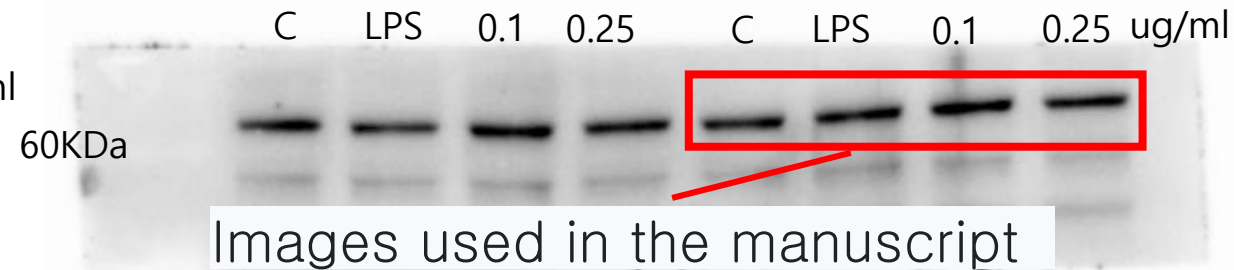

Supplement: Supplementary file 1 — Supplementary Information. [file 41598_2023_48170_MOESM1_ESM.pdf]
